# Surgical Management of Ischemic Cardiomyopathy Patients with Severe Left Ventricular Dysfunction: Is It Time to Reconsider Revascularization Surgery?

**DOI:** 10.3390/jcdd11070184

**Published:** 2024-06-21

**Authors:** Matiullah Masroor, Yixuan Wang, Chao Zhang, Nianguo Dong

**Affiliations:** 1 Department of Cardiovascular Surgery, Wuhan Union Hospital, Tongji Medical College, Huazhong University of Science and Technology, Wuhan 430022, China; dr.matiullahmasroor1@gmail.com (M.M.); wyx135791ab@hust.edu.cn (Y.W.); 2 Department of Cardiothoracic and Vascular Surgery, Amiri Medical Complex, Qargha Rd, Kabul 1010, Afghanistan

**Keywords:** ischemic cardiomyopathy, left ventricular dysfunction, myocardial viability, heart transplantation, coronary artery revascularization, surgical ventricular restoration, ventricular assist device

## Abstract

Ischemic cardiomyopathy patients with severe left ventricular dysfunction are a specific group of patients with poor surgical outcomes. There are few surgical treatment options in practice for the treatment of these patients such as heart transplantation, coronary artery bypass surgery, surgical ventricular restoration, etc. Despite multiple treatment options, there are no explicit clinical guidelines available to guide surgeons in choosing the most appropriate option and ensuring that the specific patient can benefit from the selected surgical treatment. Heart transplantation is the gold standard treatment for ischemic cardiomyopathy patients with severe left ventricular dysfunction, but it is limited to very few highly equipped centers around the world due to donor shortages, complex perioperative and surgical management, and limited technological and human resources. It is evident from some studies that heart transplant-eligible candidates can benefit from alternative surgical options such as coronary artery bypass surgery alone or combined with surgical ventricular restoration. Therefore, alternative surgical options that are used for most of the population, especially in developing and underdeveloped countries, need to be discussed to improve their outcomes. A challenge in the recent era which has yet to find a solution is to determine which heart transplant candidate can benefit from simple revascularization compared to a complex heart transplantation procedure. Myocardial viability testing was one of the most important determinants in deciding whether a patient should undergo revascularization, but its role in guiding appropriate surgical options has been challenged. This review aims to discuss the available surgical management options and their long-term outcomes for patients with ischemic cardiomyopathy, which will eventually help surgeons when choosing a surgical procedure.

## 1. Background

Cardiovascular diseases are responsible for one-third of deaths globally and ischemic heart disease (IHD) or coronary artery disease (CAD) is the leading cause of morbidity and mortality worldwide [1]. IHD is also a leading cause of heart failure (HF). According to 2020 statistics, approximately 244 million people live with IHD and about 9 million deaths are ascribed to it [2]. A proportion of patients with IHD present with severely impaired left ventricular ejection fraction (EF). Ischemic cardiomyopathy (ICM) is a condition in which the heart muscles are damaged or weakened due to the limitation of blood flow caused by CAD. In this article, an ICM patient is considered a patient with cardiomyopathy caused by CAD with an LVEF ≤ 35% with or without clinical heart failure presentation.

Ischemic etiology is considered to be a significant independent variable for mortality in cardiomyopathy patients [3]. Therefore, the management of ICM patients is a challenging task with a worse prognosis [4]. Multiple treatment options are available for ICM patients: heart transplantation (HTx) [5,6]; guideline-directed medical therapy (GDMT) alone or combined with coronary artery bypass grafting (CABG) [7,8]; surgical ventricular restoration (SVR) with or without other concomitant surgery such as mitral valve repair or replacement [9,10]; durable mechanical circulatory support such as a left ventricular assist device (LVAD) [11]; percutaneous coronary intervention (PCI) [12]; and mesenchymal stem cell transplantation [13,14,15].

The main target of ICM treatment is to re-perfuse the vulnerable myocardium when possible. Revascularization can help to halt the progression of myocardial damage by supplying adequate flow to the undersupplied myocardium and reversing the hibernating myocardium, which can subsequently increase cardiac function and prevent future ischemic events. CABG surgery for ICM patients has been documented in the literature and the results are very encouraging [16,17,18,19]. CABG combined with medical therapy has resulted in better survival, freedom from major adverse cardiovascular events (MACEs), and a low rate of repeat rehospitalization and revascularization compared to medical therapy alone [7,20,21,22] or PCI [20,22,23,24]. SVR is also a viable option for treating selected ICM patients and has resulted in promising outcomes [9].

MCS is necessary for selected patients as a bridge to transplant, bridge to recovery, or bridge to candidacy when revascularization is not feasible, or it is used as a destination therapy (DT) when revascularization and HTx are not appropriate. HTx is an established and final option in severe but suitable ICM patients from the early days even when the efficacy of CABG was doubted. These surgical options for ICM are associated with high mortality even with the development of cardiac surgery, which shows the severity and complexity of the disease [25,26]. In the presence of donor shortages and limited resources, it is important to search for alternative treatments for HTx when feasible.

There are no clear guidelines available regarding the management of ICM patients. Selecting one surgical procedure over another is a hard and crucial decision. Therefore, further studies to find factors associated with operative and follow-up mortality and overall outcomes are important for guiding clinicians in dealing with ICM patients and helping to improve the postoperative outcomes of these patients. In this article, we are focusing solely on the available surgical options for ICM patients and discussing their outcomes and long-term survival which ultimately will guide cardiac surgeons in choosing a better surgical option while treating this specific group of patients.

## 2. Heart Transplantation

HTx is an established and gold-standard treatment option for end-stage heart diseases including ischemic cardiomyopathy [5]. Due to the scarcity of donor organs and other contraindications for HTx such as advanced age, multiple comorbidities, no compliance for treatment after surgery, etc., less than 20% of transplant referrals ultimately receive a transplant [27], and therefore, alternative surgical options are adopted. In less developed and underdeveloped countries with minimum resources and no HTx facilities, alternative methods are the only options for treating these patients.

In the current era, ICM is the second leading cause of transplantation preceded by non-ICM [28]. About 30–45% of HTx patients have an ischemic etiology [28,29,30,31]. Early studies found that ischemic etiology was an independent risk factor for poor post-transplantation survival [5,29] but recent studies do not support this idea. They believe that the survival of ICM, non-ICM, or dilated cardiomyopathy (DCM) patients after HTx is comparable [6,30].

Although it is one of the main indications for heart transplantation, there is not much research available solely focusing on the outcomes and survival of ICM patients after HTx. However, some studies have compared HTx outcomes in ICM vs. non-ICM or DCM as mentioned before. There are also a few studies that compared the outcomes of HTx vs. CABG in ICM, which will be discussed in a separate section below. HTx is considered the gold standard for ICM patients but more data focusing on ICM outcomes after HTx are necessary to evaluate and compare it with other alternative procedures available for ICM.

The survival of HTx for ICM patients from the ISHLT registry from 2004 to 2014 was 84.3% at 1 year, 71.2% at 5 years, and 49.7% at 10 years [32]. Based on the evidence from the literature, the operative or 30-day mortality of HTx in ICM patients ranges from 7 to 22% [5,26,33,34,35], while 1-year survival ranges from 70 to 85%, 50 to 70% for 5-year survival, and 30 to 50% for 10-year survival in ICM patients after HTx [5,6,29,30,32].

## 3. Coronary Artery Bypass Surgery

During the early stages of CABG, it was found that patients with coronary artery disease and reduced left ventricular function have higher perioperative mortality and therefore, it was a standard not to operate on this specific group of patients. CABG was to be performed only for patients with an LVEF > 30% [26,36]. With the improvement of surgical techniques and perioperative and postoperative management, it slowly became evident that CAD patients with a severely reduced ejection fraction can benefit from CABG [18,37]. Nonetheless, CABG for this specific group remained controversial. A study by Topkara et al. analyzed 55,515 patients and categorized them based on ejection fraction. They found that low EF patients were sicker at baseline and had more than 4 times higher mortality compared to high EF patients [38]. Another study also confirmed that the perioperative morbidity in patients with a low EF was higher compared to high EF patients, resulting in longer hospital stay [17].

A 10-year follow-up of the “Surgical Treatment for Ischemic Heart Failure (STICH)” randomized clinical trial (RCT), which randomized 1212 patients with EF ≤ 35% to CABG plus medical therapy (n = 610) and medical therapy alone (n = 602), the incidence of death from any cause and cardiovascular causes and hospitalization due to cardiovascular causes were significantly less in the CABG plus medical therapy group compared to the medical therapy alone group [7]. The 5- and 10-year survival in the CABG group was 64% and 41.1%, respectively. They concluded that CABG was superior to medical therapy alone [7,25]. Nagendran et al. [24] in an RCT compared CABG (n = 1326) to PCI (n = 1599) in patients with an EF < 35%; the results after risk factor adjustment showed that CABG provided improved survival benefit and lowered the risk of repeat revascularization [24]. CABG was superior to PCI in terms of long-term survival, rehospitalization for heart failure and myocardial infarction, and subsequent revascularization [23,24]. Lee et al. [16] presented their 15-year experience of CABG in patients with an LVEF < 30% (mean 23.5%). Survival at 1, 5, and 10 years was 87.7%, 80.9%, and 44.4%, respectively, while freedom from MACEs was 96.5%, 90.3%, and 63.5% at 1, 5, and 10 years, respectively [16]. Shapira et al. [17] reported 3- and 5-year survival rates of 91 ± 3% and 76 ± 6%, respectively. Similarly, a study by Elefteriades et al. [19] had a survival of 87%, 81%, and 71% at 1, 3, and 4.5 years, respectively. The operative or hospital mortality varied in different institutions: 2.6% [17], 5.2% [19], 10.6% [18], and 11% [16].

In an RCT by Nagendran et al. [24], the independent predictors for poor long-term survival based on Cox proportional hazard analysis were age, congestive heart failure, renal failure, diabetes, peripheral vascular disease, and left main coronary artery disease [24]. Vickneson et al. found preoperative hemodynamic instability and a higher creatinine level (>166 μmol/L) as independent predictors for 1-month mortality in patients with ICM. Urgent and emergency surgery was a predictor for major adverse cardiovascular events (MACEs) and renal complications. Diabetes increased the likelihood of renal complications. In the subgroup analysis, viable myocardium was associated with a lower mortality [39]. Another study using a multivariate analysis found that advanced age was an independent predictor for in-hospital mortality while renal failure, mitral regurgitation, female gender, and postoperative respiratory complications were independent predictors of mid-term mortality [17].

The presence of angina is one of the important symptoms that surgeons consider before CABG. It is generally believed that angina indicates myocardial viability which is amenable to revascularization. A study by Cao et al. [40] in ischemic heart failure with reduced EF patients reported that perioperative mortality after CABG in patients with preoperative low myocardial viability was higher (12.5%) compared to high myocardial viability patients (3.8%) (*p* = 0.034). The 1-, 2-, and 3-year MACE-free survival rates were also significantly higher in patients with high myocardial viability. The 5- and 10-year follow-ups of the STICH trial found that patients with preoperative viable myocardium were associated with an increase in EF regardless of the treatment (either CABG or medical therapy alone), but the increase in EF did not affect survival [41,42]. They concluded that preoperative viability testing could not identify patients who would receive different survival benefits from CABG compared to medical therapy alone in patients with ICM. CABG seems to provide no additional benefit compared to medical therapy alone in ICM patients with associated right ventricular dysfunction [43].

Based on the evidence from the literature, the operative or hospital mortality in ICM patients after CABG ranges from 2 to 16% [16,17,18,19,35]. The 1-, 5-, and 10-year survival rates range from 80 to 90%, 60 to 80%, and 40 to 70%, respectively [7,16,17,19,24,25].

## 4. Heart Transplantation vs. Coronary Artery Bypass Surgery

As the two main surgical options for ICM patients, CABG and HTx procedures and their outcomes as individual procedures are discussed above. However, these studies are from different geographical areas of the world, with different patient populations and different surgical experiences of the surgeons, which can yield different outcomes. Additionally, the demographics and clinical characteristics of the cohorts are different, which can also influence the results. Although CABG and HTx are not competing procedures, to get a clear idea of their outcomes, which will subsequently help in choosing the appropriate surgical procedure for a specific patient, comparative studies with balanced preoperative risk factors between the two procedures and assessments of their short-, mid-, and long-term outcomes are essential and can address the questions posted above. To our knowledge, there are only five comparative studies in the literature comparing the outcomes of CABG and HTx in ICM patients [26,33,34,35,44]. Two of these studies by Hausmann et al. only presented the outcomes of CABG and HTx while the other three compared the results statistically as well. The basic features and information about these studies are given in Table 1.

In a study by Hausman et al., out of 265 patients who underwent CABG, 119 (44.9%) patients were younger than 60 years. Out of the 119 patients, 89 (74.8%) were referred to be candidates for HTx but underwent CABG. They were in NYHA functional classes III and IV. The postoperative outcomes of these patients were not different from those of the other CABG patients [26]. Another study by the same author analyzed the outcomes of 225 ICM patients who were referred for possible HTx but eventually underwent CABG compared to 231 ICM patients who underwent HTx. All patients of the HTx group were NYHA class III or IV before surgery, while 91.6% of the CABG group patients were in NYHA class III or IV. Of the patients who survived six months after surgery, 90.2% in the CABG group and 98.8% in HTx group could be recategorized in class I or II [34]. The main selection criteria for patients to undergo revascularization in both studies were ischemia diagnosed with myocardium thallium scintigraphy, dobutamine-induced stress echocardiography, electrocardiographic changes induced with exertion, and angina pectoris, while the criteria for referral for HTx was predominant heart failure. They concluded that CABG is a preferable surgical option for ICM patients with predominant angina, considering the high mortality and morbidity associated with immunosuppression in HTx. Contrary to their conclusion, Shum-Tim et al. [33] also conducted a comparative study in which they evaluated the survival rate, functional status, and quality of life of 14 CABG patients with 14 matched HTx patients. Initially, they evaluated 65 patients with an EF ≤ 20%; 14 were referred for transplantation but underwent CABG instead after consultation with the HTx committee. The operative mortality was the same in both groups (1/14, 7.1%). They concluded that HTx is significantly superior to CABG in postoperative quality of life and functional capacity.

A recent study published by our group compared the long-term survival of ICM patients after receiving two surgical techniques [35]. The 5-year survival was comparable between the two groups in the unadjusted cohort and propensity score-matched (PSM) cohort. In the PSM cohort, all variables were adjusted except for the EF. The mean EF in the CABG group was 31% while in the HTx group, it was 23% (*p* < 0.001). We developed a follow-up mortality risk prediction model from the values of the hazard ratio of significant variables in the multivariate Cox regression analysis. When stratified by surgical groups, CABG had an improved 5-year survival trend in the low-risk group compared to HTx, though it was not statistically significant (91.6% vs. 70.2%, *p* = 0.11), while the 5-year survival of HTx in the high-risk group was significantly higher compared to the CABG group (64.1% vs. 33.3%, *p* = 0.047) [35]. Yoon et al. [44] analyzed 1468 ICM patients categorized into four surgical intervention groups: CABG alone (n = 386), CABG + MVP (212), CABG + SVR (n = 360), and listing for HTx (n = 510). While comparing 386 CABG patients with 510 patients listed for HTx, 348 eventually underwent HTx and 94 patients died while waiting for HTx, who were included in the analysis. The 1-, 5-, and 9-year survival rates were 92%, 72%, and 53% in the CABG group and 79%, 66%, and 54% in the listing for HTx group, respectively. They also stated that the long-term outcomes of CABG and HTx listing patients were often comparable, but CABG needed viable myocardium and graftable coronaries [44]. They developed a survival prediction model for all four types of surgical procedures. To investigate whether the given treatment increased 5-year survival, they predicted the 5-year survival for each patient after each applicable therapy. Seventy-four percent of patients were eligible for CABG only, 41% for CABG+MVP, 50% for CABG + SVR, and 46% for HTx listing. In other words, 29% of the patients seem eligible for one therapy, 40% for two therapies, 24% for three therapies, and 7.5% for all four types of therapies. CABG alone and HTx listing often increased the 5-year survival rate [44].

## 5. Surgical Ventricular Restoration

Surgical ventricular restoration (SVR) is another alternative surgical option for ICM patients. SVR often includes endo-ventricular circular patch plasty (EVCPP), also known as the Dor procedure in its original or its modified form, and CABG with or without mitral valve intervention [45]. SVR can be performed in a specific group of patients that meet specific selection criteria. Usually, patients with anterior wall myocardial infarction, dilated left ventricle with large akinetic or dyskinetic segments, and ventricular dysfunction are believed to be SVR candidates [9,46]. The purpose of the procedure is to restore the physiological left ventricular volume and regain the original elliptical shape of the ventricle by resecting the scar and reconstructing the ventricle. Maxey et al. [47] found that in ICM patients with an enlarged left ventricle (end-diastolic dimension > 6.0 cm), SVR plus CABG was superior to CABG alone. In their study, the improvement in EF was significantly higher in the SVR group compared to CABG alone group (*p* < 0.01) without an increase in mortality. There was no in-hospital mortality in both groups, but late mortality was higher in the CABG group. Freedom from heart failure was achieved in 96.4% and 80% of patients in the SVR plus CABG and CABG alone groups, respectively. The combined late mortality and freedom from heart failure outcomes were significantly improved in the SVR plus CABG group (*p* < 0.05) [47]. The STICH trial randomized 1000 SVR plus CABG eligible patients to either CABG alone (n = 499) or SVR plus CABG (n = 501) and followed them for 4 years. The primary outcome was all-cause mortality and cardiac-cause rehospitalization. They found that SVR in addition to CABG can decrease the left ventricular volume compared to CABG alone, but this decrease was not associated with improvements in exercise tolerance or cardiac symptoms, or any decrease in primary outcomes in the SVR group. The improvements in exercise tolerance, cardiac symptoms, all-cause mortality, and cardiac-related hospitalization were comparable between the two groups [10].

There are some comparative studies available in the literature comparing SVR with HTx. A study published by Cotrufo et al. [48] compared the postoperative quality of life and outcomes of SVR and HTx. The SVR included EVCPP, CABG, and mitral valve surgery when necessary. The SVR group comprised 85.7% and 14.3% patients in NYHA classes III and IV while the HTx group contained 92.8% and 7.2%, respectively. The given preoperative parameters were comparable between the two groups except for age. The HTx patients were younger compared to the SVR group. The hospital mortality was 19% in the SVR group and 8.7% in the HTx group (*p* = 0.143). The 5-year survival was not significantly different between the groups, at 87.5% and 79.4% in the SVR and HTx groups, respectively. At 5 years, the freedom from heart failure rates were 93.5% and 86.2% (*p* = 0.23) and freedom from rehospitalization rates were 93.5% and 61.3% (*p* = 0.002) in SVR and HTx groups, respectively. They concluded that the selected HTx-eligible patients can be managed by SVR with satisfactory postoperative quality of life and survival outcomes [48]. Another study by William et al. [49] compared the two procedures concerning cost, length of hospital stay, NYHA status, and Kaplan–Meier survival. They found that the total length of stay and postoperative hospital stay was significantly shorter for the SVR group. The hospital and drug costs were also lower for the SVR group compared to the HTx group. At follow-up, 91% of the SVR and 98% of the HTx patients improved to NYHA classes I and II. Both procedures resulted in a significant improvement in EF and the survival was comparable between the groups. They concluded that in selected ICM patients who are eligible for HTx, SVR may be considered as a treatment option to improve graft allocation [49].

Yoon et al. [44] compared 360 SVR patients with 510 patients listing for HTx in which 348 eventually underwent HTx. Ninety-four patients in the listing group died waiting for HTx and were included in the analysis. The 1-, 5-, and 9-year survival rates were 94%, 76%, and 55% in the SVR group, and 79%, 66%, and 54% in the listing for the HTx group, respectively [44].

## 6. Durable Ventricular Assist Devices

Ventricular assist devices (VADs) are another option for treating patients with ICM. Durable VADs are now an accepted therapeutic option for end-stage heart diseases where VADs are used as a permanent therapy, referred to as destination therapy (DT) [50]. Selected ICM patients who are not candidates for CABG or HTx for various reasons could benefit from LVAD implantation. After the approval of continuous flow VAD (CF VAD) for DT, the number of VAD implantations has increased dramatically [50]. The US revised heart allocation system, which was implemented in October 2018, had a significant impact on the implantation of LVADs. The use of implantation of LVADs as DT before 2018 was about 50% while after 2018, it was above 70% [51]. As per the STS Interagency Registry for Mechanically Circulatory Support (Intermacs) 2020 annual report, which consisted of 37.1% ICM patients, the 1- and 2-year survival in isolated CF LVAD patients in recent years (2015–2019) was 82.3% and 73.1%, respectively, while the median survival was 54.6 months, which is a significant improvement compared to the 2010–2014 era [51]. The primary etiology of heart failure in the EUROMACS registry was ischemic cardiomyopathy, accounting for 40.7%. The 1-, 2-, and 3-year survival rates in isolated LVAD patients were 69%, 55%, and 44%, respectively [52]. About 40% of patients in both registries mentioned above had an ischemic etiology; further studies are needed to clarify whether the survival of ICM is worse, comparable, or better compared to non-ICM patients. To find the answer to this question and understand the outcomes of LVADs in different cardiomyopathy etiologies, Ivanov et al. studied the outcomes of ICM (60%) and DCM (40%) patients who had matched baseline characteristics and received an LVAD. They found that the in-hospital mortality and long-term survival were comparable between the groups [11].

The assumption of whether CABG in addition to an LVAD would provide some additional benefit in ICM patients was studied by Mehta et al. who compared 51 patients treated with an LVAD and 28 matched patients who received an LVAD plus CABG. The one-month survival was poor in the LVAD plus CABG group while there was no difference in ventricular arrhythmias and right ventricular failure occurrence [53]. As MCS devices are comparatively new and are still in the phase of continuous development, it will take us time to understand their long-term outcomes specifically in ICM patients. Therefore, selective and comparative studies are needed to better understand the long-term outcomes of ICM patients treated with LVADs.

## 7. Discussion

CABG and HTx are not competitive procedures but some studies have shown that patients listed for HTx or eligible HTx candidates had improved outcomes after revascularization [26,34]. Therefore, it has to be determined which group of patients from HTx-eligible candidates can benefit from CABG, which will subsequently decrease the cost, complications associated with immunosuppression, burden on the health care system, and, most importantly, optimize the donor heart utilization to some extent with the expected donor heart being used more justly for the patient with the greatest need. It has been acknowledged that it is not easy to choose between HTx and CABG in some patients [54] and the decision becomes more difficult with the recent indication of LVADs as a DT. Myocardial viability was once a determinant in helping in choosing the appropriate surgical procedures and it was supported by many previous observational studies in the past few decades [26,34], but recent findings from the STICH trial have jeopardized its integrity [41].

In the absence of clear guidelines, it is very challenging to determine which patient can benefit from revascularization or will have better or comparable outcomes with CABG compared to HTx. In the early comparative studies, which claimed that revascularization had comparable outcomes to HTx in selected patients, it had been emphasized that CABG should be performed in patients with a viable myocardium and signs of ischemia. Viability assessment by noninvasive stress imaging (CMR, SPECT, PET, and stress echo) for ICM patients deemed eligible for revascularization achieved IIb recommendation status in the 2018 ESC/EACTS guidelines [55]. Studies showed that 37–81% of ICM patients have a viable myocardium [42,56,57]. The viability phenomenon has been challenged after the findings of the STICH trial, which indicate that preoperative myocardial viability cannot differentiate the patients that will benefit more from CABG compared to optimal medical therapy. They believe that a preoperative viable myocardium can improve postoperative EF irrespective of the treatment option but that the increase in EF is not associated with improved survival [41]. Liga et al. [58] in a systematic review and meta-analysis of randomized controlled trials concluded that ischemia and viability testing do not seem to be mandatory and do not help in treatment guidance [58].

These controversial findings compel us to rethink the idea of viability and find out exactly what degree of viable or scarred myocardium can undergo revascularization. The 2018 ESC/EACTS guidelines for EF ≤ 35% and chronic heart failure patients recommend CABG as a Class I revascularization strategy in multivessel disease and acceptable surgical risk patients [55]. There is also a consensus published in 2021 by the American Association for Thoracic Surgery for the therapeutic management of patients with ICM and heart failure, which categorically does not focus on HTx and dVADs but rather focuses on CABG, PCI, and medical management in ICM patients [46]. CABG and HTx are two different surgical procedures aimed at different patients, but the eligible candidates are very similar when it comes to ICM patients where the root cause is the same. There are detailed listing criteria available for HTx patients [59], but how many of those ICM patients eligible for HTx can benefit from surgical revascularization is a real question to be answered.

There should be clear, strict, and elaborated guidelines from cardiothoracic and vascular societies for ICM patients to identify those who can benefit from CABG instead of HTx. Having a specific guideline in place can encourage surgeons to adopt a more homogenous approach. Implementing these guidelines can provide more evidence in coming years when the short-, mid-, and long-term outcomes are available and then the guidelines can be modified according to the results of the studies. If the reason for the lack of guidelines is the paucity of evidence in the literature, then there should be a consensus from world experts based on the six decades of experience in the field of HTx and CABG.

So, in this era of ambiguity of whether or not viability plays a role in postoperative outcomes, the identification of new reliable variables and factors including biomarkers that can predict postoperative outcomes after CABG will be a great achievement. A study evaluated myocardial scar severity by late gadolinium enhancement cardiac magnetic resonance imaging (LGE-CMR) and found that patients with a >75% transmural scar in less than six segments had a significantly lower risk of cardiac death after CABG compared to medical therapy while patients with a >75% transmural myocardial scar in more than six segments had no significant difference regarding cardiac mortality between the two groups [60]. Some researchers believe that the results of the STICH trial may be influenced by using SPECT and DSE which have suboptimal image resolution and could not distinguish between hibernating and scarred myocardium. They believe measuring myocardial viability with PET and CMR could precisely determine metabolically active myocardium and quantify myocardial scars [61].

Another way of aiding the surgical decision may be the use of risk scores in ICM patients. As discussed previously, in our recent study, high-risk patients had superior survival with HTx while low-risk patients had a better trend with CABG [35]. A system developed by Yoon et al. can also play an important role in detecting which surgical procedure best suits a specific patient and what would be the survival if an alternative procedure is adopted [44]. The development of risk prediction models and systems based on using the patients’ demographics, imaging results, and preoperative lab data to stratify patients into risk groups and predict outcomes would pave the way for adopting the appropriate surgical approach in clinical practice.

## 8. Conclusions

A standard approach towards surgically managing ischemic cardiomyopathy patients with severe left ventricular dysfunction is very important. Heart transplantation is the gold standard option for ischemic cardiomyopathy patients with severe left ventricular dysfunction and predominant heart failure, but it is not always possible due to multiple reasons. Therefore, alternative options such as CABG alone or CABG with surgical ventricular restoration should be considered. Explicit guidelines or a consensus should be established that can explain the role of myocardial viability and extent of ischemia, which are amenable to revascularization, to guide surgeons’ decisions when choosing surgical options, and to identify eligible heart transplant candidates who can benefit from coronary revascularization. Until the establishment of guidelines, the viability testing with PET and CMR can be trusted. The surgical choice should be made by a multidisciplinary team considering the myocardial viability and scar severity, coronary anatomy, eligibility for transplantation, feasibility of the procedure, benefit and risk assessments, and the available facilities.

## Figures and Tables

**Table 1:** Comparative studies of coronary artery bypass surgery versus heart transplantation in patients with ischemic cardiomyopathy and left ventricular dysfunction (LVEF ≤ 35%).

Authors	Country	Year of Publication	Study Duration	No. of pts		Pre-Op EF		Operative/30-Day Mortality		Follow Up	Survival	
				CABG	HTx	CABG	HTx	CABG	HTX		CABG	HTX
Hausmann et al. [26]	Germany	1994	1986–1992 1990–1992	265	55	10–30% mean 24%	9–30% mean 21%	7.60%	21.80%	Mean: 24.1 months	89.1% at 1 year 87.8% at 2 years 86.9% at 3 years 85.6% at 4 years	68.9% at 1 year 66.3% at 2 years
Hausmann et al. [34]	Germany	1997	1986–1994	225	231	mean 23%	mean 21%	7.10%	18.20%	36.5 months	90.8% at 2 years 87.6% at 4 years 78.9% at 6 years	74.9% at 2 years 73.2% at 4 years 68.9% at 6 years
Shum-Tim et al. [33]	Canada	1999	1991–1994	14	14	less or equal to 20%	less or equal to 20%	7.10%	7.10%	Mean: 20 months (6 months—4 years)	71.40%	NA
Yoon et al. [44] *	USA	2010	1997–2007	386	510	22 ± 4.2%	16 ± 7.6%	7/386 (1.8%)	18/348 (5.2%)	Mean: 3.8 + 2.8 years	92% at 1 year; 82% at 3 years 72% at 5 years 53% at 9 years	79% at 1 year 72% at 3 years 66% at 5 years 54% at 9 years
Zhang et al. [35]	China	2024	2011–2021	106, After PSM 51	112, After PSM 51	31.28 ± 3.28	23.44 ± 7.09	14.15%, After PSM 19.61%	10.71%, After PSM 15.69%	Median: 48 months	83% at 1 year 80.7% at 3 years 73.3% at 5 years After PSM: 78.3% at 1 year 76.2% at 3 years 70.6% at 5 years	80.3% at 1 year 76.5% at 3 years 73.1% at 5 years After PSM: 76.4% at 1 year 72.3% at 3 years 66.2% at 5 years

* In this study HTx patients referred to the HTx listing patients, 94/510 patients in the HTx group died while waiting for HTx and were included in the survival analysis. CABG: coronary artery bypass grafting; HTx: heart transplantation; PSM: propensity score matching.

## Data Availability

Not applicable.
